# Aryl Hydrocarbon Receptor (AHR) Suppresses Arsenic (As^3+^)-Induced Malignant Transformation by Antagonizing TOX Expression

**DOI:** 10.7150/ijbs.107268

**Published:** 2025-03-31

**Authors:** Wenxuan Zhang, Ziwei Wang, Yao Fu, Chitra Thakur, Haoyan Ji, Zhuoyue Bi, Yiran Qiu, Millie Elangbam, John Haley, Fei Chen

**Affiliations:** Stony Brook Cancer Center, Department of Pathology, Renaissance School of Medicine, Stony Brook University. Lauterbur Drive, Stony Brook, NY 11794, USA.

**Keywords:** Arsenic, Carcinogenesis, Cancer stem-like cells, AHR, TOX

## Abstract

Environmental arsenic (As³⁺) exposure poses a significant public health concern due to its carcinogenic potential. Our previous research suggests that As³⁺-induced carcinogenesis is mediated by inhibition of the aryl hydrocarbon receptor (AHR). However, the precise role of AHR in As³⁺-induced malignant transformation as well as cancer stem-like cell (CSC) formation, along with its underlying mechanisms, remains unclear. In this study, we used BEAS-2B cells with CRISPR-Cas9 gene editing, RNA sequencing, and immunoprecipitation to examine AHR's role in As³⁺-induced CSC development. Our findings reveal that AHR suppresses CSC formation triggered by low-dose As³⁺ (0.5 μM) via transcriptional repression of TOX, a high mobility group box DNA binding protein that play a critical role in T cell exhaustion within tumor immunology. *TOX* knockdown inhibited CSC formation, while its overexpression enhanced cMYC, a CSC-associated transcription factor. TOX interactome analysis identified associations with proteins such as KCTD10, TRIM21, HMGA1, FLOT1, and FLOT2, which may regulate TOX's stability and activity. Enrichment analyses highlighted their involvement in cancer-related pathways, supporting the role of TOX in promoting CSC formation during As³⁺-induced carcinogenesis. Notably, this study identifies TOX as an oncogenic factor in non-immunological contexts and underscores AHR's tumor-suppressive function through TOX repression, offering novel insights into the mechanisms underlying As³⁺-induced carcinogenesis.

## Introduction

Arsenic is a well-known human carcinogen with a strong association with various cancers, particularly those affecting the skin, lungs, bladder, liver, and kidneys 1. Its carcinogenicity is primarily attributed to long-term exposure to inorganic arsenic (iAs), especially arsenite (As³⁺), which commonly occurs through contaminated drinking water, occupational exposure, and certain food sources 2. Unlike many carcinogens, As³⁺ does not directly damage DNA; rather, it promotes carcinogenesis through indirect mechanisms, including oxidative stress, epigenetic modifications, inflammation, disruption of cellular signaling pathways (p53 and NF-κB), and alterations of cell cycle and apoptosis 3, 4. Research into As³⁺ toxicity has informed regulatory measures aimed at limiting human exposure, particularly in drinking water, which is often contaminated in areas with natural mineral deposits. Many countries have established maximum allowable limits for arsenic in drinking water to mitigate exposure risks. For instance, the U.S. Environmental Protection Agency (EPA) has revised the maximum contaminant level (MCL) for arsenic in drinking water from 10 to 50 ppb 5. Despite these regulations, EPA estimates indicate that the lifetime risk of developing cancer from consuming drinking water containing arsenic at 10 ppb remains significant, with an estimated risk of about 1 in 500 people 6. This risk surpasses that associated with other common environmental pollutants, including hexavalent chromium, cadmium, and Per- and Polyfluoroalkyl Substances (PFAS).

Cancer stem-like cells (CSCs) are a driving force behind long-term growth, therapeutic resistance, metastasis, and recurrence 7. CSCs can originate either from the blocked differentiation of normal stem or progenitor cells or from the dedifferentiation of cancerous or normal cells 8. The involvement of As³⁺ in the induction of CSCs was first suggested by studies on fetal As³⁺ exposure and skin cancer in v-Ha-Ras transgenic mice (Tg.AC) 9. Fetal exposure of As³⁺ sensitized these mice to 12-O-tetradecanoylphorbol-13-acetate (TPA)-induced skin malignancies and upregulated the expression of *CD34* and *Rac1*, two genes crucial for keratinocyte stem cells and skin CSCs. Using non-cancerous epithelial stem-like cells derived from human prostate tissue, Tokar et al. demonstrated the possible conversion of normal stem cells to CSCs in response to long-term As³⁺ exposure 10. However, it remains unclear whether this conversion results from inhibition of differentiation of the normal stem cells or direct activation of oncogenic signals by As³⁺. Other studies suggest that As³⁺ activates Wnt signaling and induces epithelial to mesenchymal transition (EMT), a process in which epithelial cells lose polarity while acquiring migratory and invasive properties 11. EMT is closely associated with the acquisition of stem-like traits, which contributes to CSC formation 12. By promoting EMT, As³⁺ facilitates tumor invasion and generates cells with enhanced self-renewal and resistance capabilities 13. Our previous report showed the induction of CSCs following the consecutive treatment of human bronchial epithelial cells (BEAS-2B) with environmentally relevant concentrations of As³⁺ over six months 14. These As³⁺-induced CSCs exhibited increased expression of key stemness transcription factors, including OCT4, SOX2, KLF4, and MYC 15, 16. In addition to their tumorigenicity in mice, they demonstrated characteristics of self-renewal both *in vitro* and *in vivo*, as well as resistance to chemotherapy-induced apoptosis. However, the precise mechanisms underlying As³⁺-induced CSC generation remain poorly understood.

Aryl Hydrocarbon Receptor (AHR), the first identified chemical carcinogen-activated transcription factor, is a member of the basic helix-loop-helix (bHLH) family. Initially linked to carcinogenesis in response to exogenous chemicals like 2,3,7,8-tetrachlorodibenzo-p-dioxin (TCDD), AHR has recently gained recognition as a crucial modulator of various biological processes, including immune response, inflammatory diseases, and the carcinogenicity of environmental chemicals 17. Emerging evidence suggests that AHR plays a significant role in stem cells and CSCs 18. Some reports indicate that activated AHR protects intestinal stem cells from genotoxic stress and promotes CSC phenotypes while facilitating metastasis 19. Conversely, other studies have reported opposing effects of activated AHR on CSC development and renewal. For instance, Contador-Troca et al. demonstrated that AHR inhibits tumorigenesis and metastasis in melanoma by antagonizing the expression of the cancer stem cell-associated enzyme aldehyde dehydrogenase (ALDH1A1) 20. This inverse correlation between AHR activation and the CSC phenotype has also been observed in prostate cancer and leukemia 21-23. Moreover, mounting evidence over the past few years has suggested a tumor-suppressive nature of AHR in both cellular and animal tumor models 17. In our recent studies on environmental As³⁺-induced malignant transformation, biochemical analyses and ChIP-seq data suggested that AHR may exert tumor-suppressive effects by antagonizing TGF-β and Nrf2 signaling pathways 15. However, the specific role of AHR in the generation of CSCs induced by As³⁺ remains unclear, and the underlying mechanisms warrant further investigation.

TOX (thymocyte selection-associated high mobility group box) is a nuclear DNA-binding factor belonging to the high-mobility group box superfamily, which binds DNA in a sequence-independent but structure-dependent manner 24. The TOX family comprises four subfamily members (TOX1-4, with TOX1 also known as TOX) and serves as a critical transcription factor implicated in the development of malignancies, particularly those involving T cells and other lymphocytes 25. Previous studies suggest that TOX contributes to cancer progression and influences therapeutic outcomes by regulating the tumor microenvironment and promoting T cell exhaustion 26, 27. In this report, we present evidence demonstrating that AHR antagonizes As³⁺-induced TOX expression. Data derived from immunohistochemistry, RNA sequencing (RNA-seq), ChIP sequencing (ChIP-seq), and immunoprecipitation reveal that AHR knockout facilitates As³⁺-induced malignant transformation, accompanied by the de-repression of TOX. These findings provide new insights into the mechanisms by which As³⁺ drives CSC formation and underscore the potential therapeutic value of targeting AHR and TOX in cancer treatment.

## Materials and Methods

### Data mining

We analyzed open-access genomic and transcriptomic datasets of lung cancer patients from UCSC Xena and TCGA. Differential expression of AHR in normal lung tissues and primary tumors was calculated using GENT2, a platform for exploring gene expression patterns across normal and tumor tissues 28. The analysis considered parameters such as pathological subtypes of lung cancer (lung cancer, lung adenocarcinoma, and lung squamous cell carcinoma), subtypes of lung adenocarcinoma, and metastasis. Comparison of AHR gene expression among normal, tumor, and metastatic tissues was performed using TNMplot 29. Additionally, a Kaplan-Meier survival analysis was conducted using a database containing survival information for 2,166 lung cancer patients along with corresponding gene expression data. The probe set *202820_at*, which detects the open-reading frame (ORF) of AHR mRNA, was selected as the optimal probe. Survival curves with *p*-values < 0.05 between groups with higher AHR (-high) and lower AHR (-low) expression levels were considered significantly different.

### Immunohistochemistry of human lung cancer tissue microarray

Lung cancer tissue microarray BC041115e and lung carcinoma with matched lymph node metastatic carcinoma tissue microarray LC817b were purchased from USBiomax (Rockville, MD). Immunohistochemical staining (IHC) for AHR was performed on the tissue microarray slides as previously described 15. Briefly, paraffin-embedded tissue sections underwent deparaffinization, rehydration, blocking of endogenous peroxidase activity, and antigen retrieval in sequence. The slides were then treated with 5% goat serum, followed by incubation with primary antibody against AHR (Enzo Life Sciences, cat#PSC-15-576, diluted: 1:100) overnight at 4 °C. This was followed by incubation with a goat anti-rabbit IgG antibody (Sigma-Aldrich, cat#12-448, diluted: 1:200) at room temperature for 2 h. The slides were subsequently treated with the ABC reagent (Vectastatin Elite ABC kit, cat#PK-6100), and the chromogen was developed using diaminobenzidine (DAB). Nuclei were counterstained with hematoxylin (Sigma-Aldrich, St. Louis, MO). Imaging was performed under the bright field of a Nikon Eclipse Ti-S Inverted microscope (Mager Scientific, Dexter, MI, USA), and the results were analyzed using Nikon's NIS Elements BR 3.2 software. Absence of staining in 10 randomly selected images was considered negative for IHC, while the presence of more than 50% signal intensity of DAB staining in all 10 images was scored as positive.

### Cell culture

The human bronchial epithelial cell line BEAS-2B was obtained from ATCC (Manassas, VA). BEAS-2B cells were cultured in Dulbecco's Modified Eagle's Medium-high glucose medium (DMEM) (Sigma-Aldrich, cat#11965092), supplemented with 5% (v/v) fetal bovine serum (FBS) (R&D, cat#S11150), 1% (v/v) penicillin-streptomycin (Gibco, cat#15140122) and 1% (v/v) L-Glutamine (Gibco, cat#25030081). Cells were maintained at 37℃ in a humidified incubator with 5% CO_2_.

### Generation of AHR knockout cell lines

AHR knockout in BEAS-2B cells was achieved using CRISPR-Cas9 gene editing technology. Guide RNAs (sgRNAs) for AHR knockout were designed with the CHOPCHOP online tool (uib.no). Two sgRNAs were selected, targeting exon 1 (sgRNA-1: 5'-TCACCTACGCCAGTCGCAAGCGG-3') and exon 2 (sgRNA-2: 5'-AGCGGCATAGAGACCGACTT-3') of the AHR gene. These sgRNAs and their reverse complementary strands were synthesized by IDT (Newark, NJ), annealed at 95°C for 5 min, and cooled to 25°C at a rate of 5°C/min. The annealed sgRNAs were inserted into a linearized pSpCas9-2A-Blast plasmid (Addgene, cat#118055), which was digested with the restriction enzyme Bpil (Thermo Fisher Scientific, cat# FD1314). The ligation product was then transformed into competent *E. coli* DH5α cells (Thermo Fisher Scientific, cat#EC0112) following the manufacturer's protocol. Plasmids were extracted using the QIAprep Spin Miniprep Kit (Qiagen, cat#27104) and sequenced by Genewiz (South Plainfield, NJ, USA) to confirm the correct insertion of sgRNAs. BEAS-2B cells were seeded at a density of 2 x 10⁵ cells per well in a 6-well plate and incubated overnight. For transfection, 500 ng of the CRISPR plasmid, 200 µl of Opti-MEM I reduced-serum medium (Thermo Fisher Scientific, cat#31985070), and 1.5 µl of Lipofectamine 2000 (Invitrogen, cat#11668027) were combined and incubated for 5 min at room temperature to form the DNA-lipid complex. The complex was then added to each well, following the manufacturer's protocol. After 48 h, the cells were transferred to a 10-cm dish and cultured in the presence of 10 µg/ml Blasticidin (Gibco, cat#A1113903) for 2 weeks to select for resistant colonies. Colonies were isolated under a microscope, and western blotting was used to screen for AHR expression. Colonies without AHR expression were designated as knockout (KO) cells, while those with AHR expression were designated as wild-type (WT) cells.

### As^3+^-induced malignant transformation and soft agar clone formation assay

Transformed BEAS-2B cell lines (Trans-WT and Trans-AHR KO) were established by treating WT and AHR KO cells with 0.5 µM As^3+^ for 26 weeks. The culture medium was refreshed every other day, with cells passaged weekly. In parallel, untreated WT and AHR KO cells were maintained as parental controls. Soft agar assays were conducted in 6-well plates using a two-layer solid medium. The bottom layer was prepared by mixing 1% Difco agar (BD, cat#214010) with DMEM and FBS to create a 0.5% agar solution, which was added to each well and allowed to solidify at room temperature. The top layer consisted of 0.33% agar, made by combining 0.66% Difco agar with DMEM and FBS, then mixed with 5000 cells and placed over the solidified bottom layer. After a 4-week incubation, clones were visualized with 200 µl of MTT solution (1 mg/ml), and colonies were counted. Images of individual colonies were captured using an EVOS M7000 microscope.

### Western Blotting

Protein extraction was performed using 1X RIPA buffer (Cell Signaling Technology, cat#9806) supplemented with protease and phosphatase inhibitor cocktail (Thermo Fisher Scientific, cat#A32957) and PMSF (Thermo Fisher Scientific, cat#36978). Protein concentrations were measured using the BCA Protein Assay Reagent Kit (Thermo Fisher Scientific, cat#23225). Samples were prepared with 4X LDS sample buffer (Thermo Fisher Scientific, cat#NP0008) and dithiothreitol (Thermo Fisher Scientific, cat#R0861), then denatured at 95°C for 10 min. Proteins were separated on a 10% SDS-PAGE gel and transferred onto a PVDF membrane (Millipore). Membranes were blocked with 5% non-fat milk for 1 h at room temperature, followed by overnight incubation with primary antibodies at 4°C. The following day, the membranes were washed three times for 10 min each and incubated with the appropriate secondary antibody for 1 h at room temperature. Bands were visualized using the ChemiDoc MP Imaging System (Bio-Rad). Antibodies used included anti-PD-L1(CST, cat#13684, 1:1000), anti-cMYC (CST, cat#9402, 1:1000), anti-AHR (ENZO, cat#PSC-15-576, 1:1000), anti-TOX (Thermo Fisher Scientific, cat#PA5-111709), anti-β-Tublin (CST, cat#2128, 1:1000), anti-H3 (CST, cat#9715, 1:1000), anti-KLF4 (CST, cat#4038, 1:1000), anti-OCT4 (CST, cat#2750, 1:1000), anti-SOX2 (CST, cat#2748, 1:1000), anti-NANOG (CST, cat#3580, 1:1000), anti-KCTD10 (Sigma, cat#HPA014273, 1:1000), anti-TRIM21 (Proteintech, cat#27279-1-AP, 1:1000), anti-HMGA1 (Abcam, cat#ab252930, 1:1000), anti-FLOT1 (Abcam, cat#ab133497, 1:1000), anti-FLOT2 (Abcam, cat#ab307422, 1:1000), anti-GFP (Abcam, cat#ab290, 1:1000), and anti-Ubiquitin (CST, cat#8240, 1:1000).

### Quantitative real-time PCR (qPCR)

Total RNA was extracted using the RNeasy Plus Mini Kit (Qiagen, cat#74104), and RNA concentration was measured with a NanoDrop spectrophotometer (Thermo Fisher Scientific, Waltham, MA). For cDNA synthesis, 1 μg of RNA was reverse-transcribed using the High-Capacity cDNA Reverse Transcription Kit (Applied Biosystems, cat#2616251). Real-time PCR was performed using the Fast SYBR Green Master Mix (Applied Biosystems, cat#2211538) on a QuantStudio 7 instrument (Applied Biosystems). GAPDH served as an internal control, and the relative mRNA expression levels were calculated using the 2^-ΔΔCt^ method.

### RNA sequencing and bioinformatics analysis

Total RNA was extracted from cells using the RNeasy Mini Kit (Qiagen, cat#74104) and prepared for sequencing with the TruSeq Stranded mRNA Library Kit (Illumina). Libraries were sequenced on an Illumina NextSeq 500 system, and raw reads were filtered and aligned to the Homo sapiens reference genome (GRCh38/hg38). Gene expression levels were quantified as FPKM (Fragments Per Kilobase Million) values. Differential gene expressions were analyzed through pairwise comparison, with genes exhibiting a fold change > 1.2 and *p*-value < 0.05 identified as differentially expressed genes (DEGs). Functional enrichment analyses, including KEGG, Gene Ontology (GO), and Gene Set Enrichment Analysis (GSEA), were performed in R using the clusterProfiler package (v4.12.0), with significant pathways and terms defined as those with *p*-values < 0.05.

### Nuclear Protein Extraction

Cells were seeded and incubated overnight, followed by As³⁺ treatment for 6 h. Nuclear and cytoplasmic protein fractions were isolated using the Nuclear Extraction Kit (Abcam, Cat#ab221978) according to the manufacturer's instructions. Protein expression was analyzed by Western blotting, as previously described. Histone H3 and tubulin served as internal controls for nuclear and cytoplasmic proteins, respectively.

### Immunofluorescence staining

Cells were seeded on 2-well slides (Ibidi, cat#80286) and incubated overnight. Following a 6 h As³⁺ treatment, cells were fixed and blocked. Subsequently, cells were incubated with anti-TOX antibody (Thermo Fisher Scientific, cat#PA5-111709) overnight at 4°C, followed by secondary antibody for 1 h at room temperature. After each incubation, slides were washed three times with PBS. A drop of DAPI (Abcam) was applied for nuclear staining, and slides were mounted for imaging. All samples were examined and photographed using an EVOS M7000 microscope (Invitrogen).

### Co-Immunoprecipitation (co-IP)

Cells were seeded in a 75-cm² flask and incubated overnight. Upon reaching > 80% confluence, cells were digested with 0.05% trypsin (Gibco, cat#25300062) and centrifuged at 1000 rpm for 5 min. After aspirating the supernatant, the cell pellet was lysed with 500 μl of ice-cold lysis buffer (1X RIPA with protease inhibitors) for 30 min and sonicated on ice (10 pulses). The lysate was then centrifuged, and protein concentration was determined via BCA assay. For IP, 1 mg of total protein was incubated with 1 μl of anti-GFP antibody (Abcam, cat#ab290) or 1 μl of anti-IgG control antibody (Abcam, cat#ab172730) at 4°C overnight. Immune complexes were pulled down using the Classic Magnetic IP/Co-IP Kit (Thermo Scientific, cat#88804) and eluted under low-pH conditions to separate the beads from the supernatant. Protein levels were subsequently analyzed by Western blotting.

### Liquid chromatography with tandem mass spectrometry (LC-MS/MS)

Protein complexes bound to the beads were washed twice with ice-cold TBS, then transferred to a fresh tube and washed once with TBS and once with ice-cold pure water. The beads were suspended in 100 µl of 100 mM ammonium bicarbonate (pH = 8) containing 5 mM DTT and heated at 90°C for 20 min. Following the heating step, cysteines were alkylated by adding 10 mM iodoacetamide, and proteins were digested with trypsin at 37°C. Peptides were then desalted, dried, and resuspended in 0.1% formic acid in water prior to LC-MS/MS analysis. Peptide abundance values were obtained using an Orbitrap instrument (Thermo Fisher Scientific, Q-Exactive HF), followed by protein database searching. HPLC C18 columns were prepared using a P-2000 laser puller (Sutter Instruments) and silica tubing (100 µm ID x ~20 cm). Peptides were separated on the resolving column at a flow rate of 300 nL/min. Electrospray ionization was performed with a spray voltage of 2.3 kV. Parent ions with charge states of 2+, 3+, and 4+ were selected with a 15-second exclusion period. Data acquisition was carried out using Xcalibur software (Thermo Fisher Scientific). Raw data were analyzed using Proteome Discoverer v2.2 software (Thermo Fisher Scientific) employing label-free quantitation. The resolution of MS data searches was set to 10 ppm and 0.05 Da, respectively. Protein identifications were binned at < 1% and < 5% FDR cutoffs. The human Uniprot database (639,722 entries) was used for data alignment. Fold change ratios were obtained through matched peptide-based label-free quantitation, and *p*-values were calculated using the Benjamini-Hochberg correction for False Discovery Rate (FDR).

### Cell transduction

Lentiviral particles used in this study were purchased from Origene (Rockville, MD). The sequences of the lentiviral shRNA particles for *TOX* knockdown (cat#TL315697V) are as follows: TOX-shRNA#1: CTCACCATCTCCACCTGGAAGCAAGTCTG; TOX-shRNA#2: CCAGTCACAGCTAAGTGCTCAACTTGGTT; TOX-shRNA#3: CAAGCCGAATAACCAAATGCCAGTGACTG; TOX-shRNA#4: CCGCCTCTTCACCAGCATCTCAACATGCA. Additionally, a lentiviral shRNA scramble control particle was obtained from Origene (cat#TR30021V). To establish *TOX* overexpression cell lines with a GFP tag in AHR KO cells, we utilized the TOX-mGFP tagged ORF clone lentiviral particle (cat#RC203792L4V) and Lentiviral ORF particles of pLenti-C-mGFP-P2A-Puro (cat#PS100093V). For cell transduction, 5 x 10^5^ cells were seeded into 24-well plates overnight. The lentiviral particles were transfected using TransDux MAX (SBI, cat#LV806A-1) according to the manufacturer's protocol. After 72 h, 0.5 µg/ml puromycin (Gibco, cat#A1113803) was added to the culture medium for selection. The efficacy of knockdown and overexpression was assessed by Western blotting and qPCR.

### Statistical analysis

Statistical significance of all quantitative data was assessed using Student's t-test and one-way ANOVA, with results expressed as mean ± standard deviation (SD). All statistical analyses were two-sided, and a significance level of *p* < 0.05 was established. GraphPad Prism 9 software was utilized for all statistical analyses.

## Results

### AHR as a tumor suppressor and prognostic marker in lung cancer

**Figure 1:**
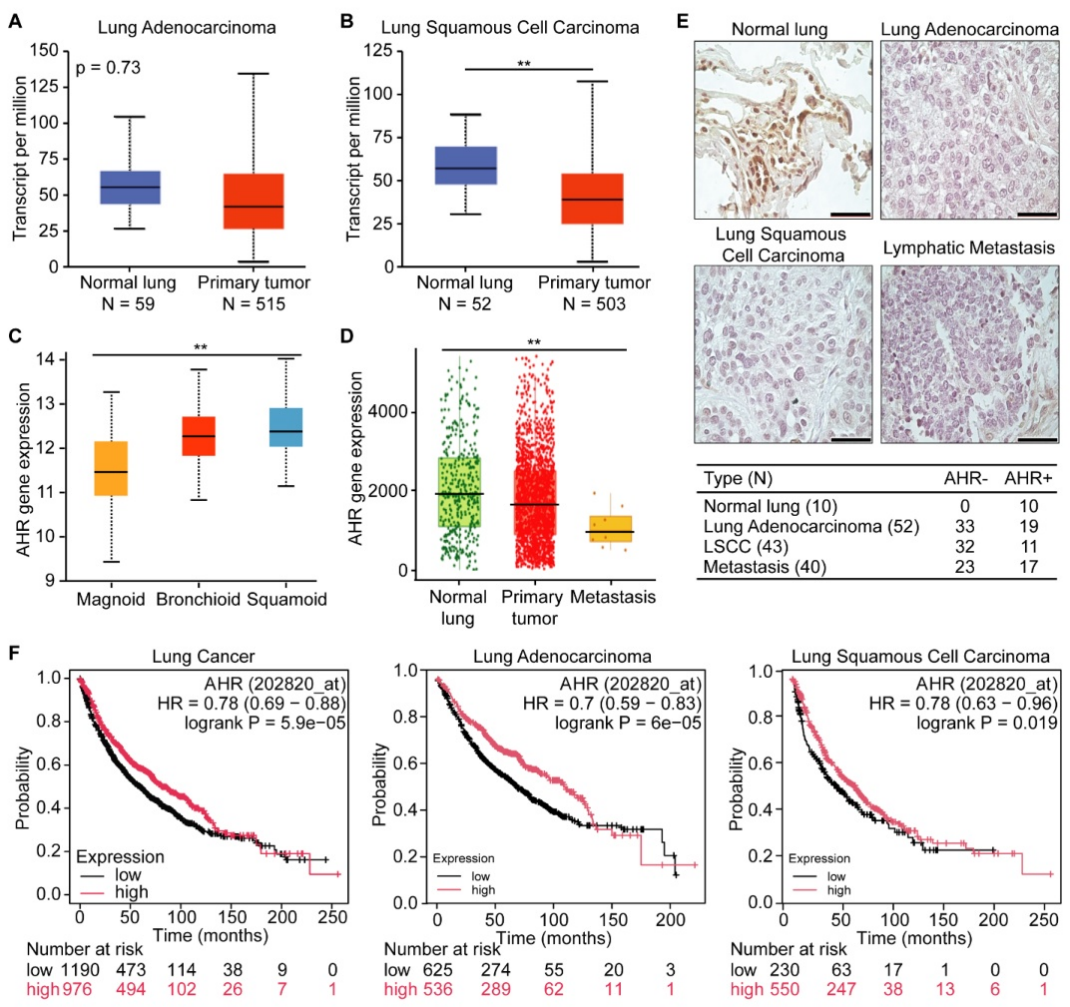
AHR functions as a tumor suppressor in lung cancer.** A**: *AHR* expression in lung adenocarcinoma and normal lung tissue. **B**: *AHR* expression in lung squamous cell carcinoma and normal lung tissue. **C**: *AHR* expression across different subtypes of lung adenocarcinoma (magnoid, bronchioid, and suqamoid). **D**: *AHR* expression in normal lung (n = 3,691), primary tumor (n = 29,376) and metastasis (n = 453). **E**: Representative immunohistochemical staining images of AHR in normal lung, lung adenocarcinoma, lung squamous cell carcinoma (LSCC), and lymphatic metastasis, with quantification of AHR-positive samples. **F**: Kaplan-Meier survival plots for lung cancer patients, categorized by lung cancer types (lung cancer, lung adenocarcinoma, and lung squamous cell carcinoma) with low or high *AHR* expression. Higher *AHR* expression is associated with improved overall survival in lung cancer. HR: Hazard Ratio.

To assess *AHR* expression in human lung cancers, we analyzed data from the publicly available TCGA lung cancer dataset. We found that *AHR* expression was significantly lower in primary tumors compared to normal tissue in both adenocarcinoma and squamous cell carcinoma (**Figures 1A-B**). We further examined *AHR* expression across different lung cancer subtypes and metastatic tissues. Lung adenocarcinomas are categorized into three molecular subtypes: bronchoid, magnoid, and squamoid 30. The bronchoid subtype, often observed in nonsmoking female patients, is associated with well-differentiated histology, *EGFR* mutations, and more favorable survival outcomes. In contrast, the magnoid and squamoid subtypes are typically linked to advanced disease and heavy smoking exposure, with magnoid tumors demonstrating the poorest survival due to frequent *KRAS*, *TP53*, and *STK11* mutations 31. Our data mining results indicated that *AHR* expression ranks lowest in the magnoid subtype, followed by bronchoid and squamoid (**Figure 1C**). When comparing *AHR* expression levels in normal tissue, primary tumors, and metastatic sites, we observed the lowest *AHR* expression in metastases, followed by tumor tissues (**Figure 1D**). To directly support these observations, we performed AHR immunohistochemistry on tissue microarray slides BC041115e and LC817b. The BC041115e array comprises 120 cases, including squamous cell carcinoma, adenosquamous carcinoma, adenocarcinoma, large cell carcinoma, small cell carcinoma, carcinoid, and normal lung tissues. The LC817b array includes 40 paired lung cancer samples with matched lymph node metastases. The percentage of AHR-positive cases was significantly higher in normal lung tissue (10/10) compared to cancer tissue (47/135) (**Figure 1E**). To further explore AHR's prognostic value in lung cancer, we conducted survival analyses. Kaplan-Meier survival curves showed that high *AHR* expression is associated with improved overall survival in lung cancer patients (**Figure 1F**). Together, these findings suggest that increased AHR expression correlates with favorable outcomes in lung cancer and may serve as a beneficial prognostic factor in aggressive cases.

### AHR suppresses As^3+^-induced malignant transformation in BEAS-2B cells

**Figure 2:**
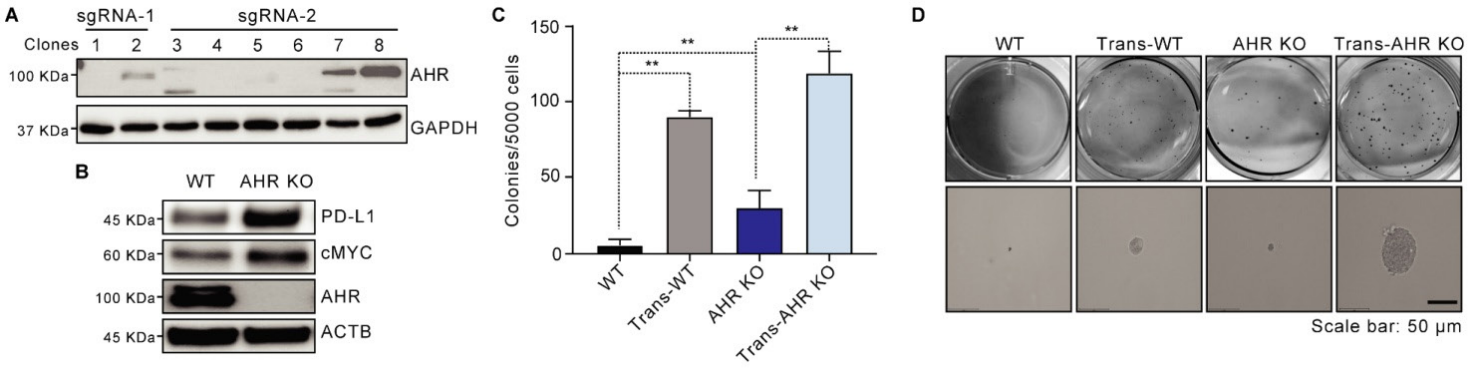
AHR inhibits As^3+^-induced malignant transformation in BEAS-2B cells. **A**: Western bolting image showing the efficiency of AHR knockout. **B**: Immunoblots demonstrating the expression of PD-L1, cMYC, AHR, and ACTB in WT and AHR KO cells. **C**: Soft agar colony formation assays of WT, Trans-WT, AHR KO, and Trans-AHR KO cells. Data are presented as mean ± SD, n = 6, **p < 0.01 vs. control. **D**: Representative images of colony morphology from soft agar assay.

To investigate AHR's role in As³⁺-induced malignant transformation, we generated an AHR KO cell line using BEAS-2B cells through CRISPR-Cas9 gene editing. The knockout efficiency was validated in clones, with clone 1, which lacked AHR expression, designated as AHR KO, and clone 8 serving as the wildtype (WT) (**Figure 2A**). Consistent with prior findings that AHR knockout impacts stemness-associated proteins, Western blotting revealed increased PD-L1 and cMYC levels in AHR KO cells compared to WT cells (**Figure 2B**). We then exposed both WT and AHR KO cells to 0.5 μM As³⁺ for 26 weeks to model chronic environmental exposure. Soft agar assays demonstrated a significant increase in colony formation in WT cells following As^3+^ treatment, with a 15.26-fold increase in colony frequency compared to untreated controls (**Figure 2C-D**). Notably, AHR knockout further enhanced anchorage-independent growth in BEAS-2B cells, with As³⁺ exposure increasing colony formation frequency by 20.13-fold compared to WT cells (**Figure 2C-D**), further reinforcing AHR's tumor-suppressive role in As³⁺-induced malignant transformation.

### AHR as a repressive transcription factor regulates TOX expression

**Figure 3:**
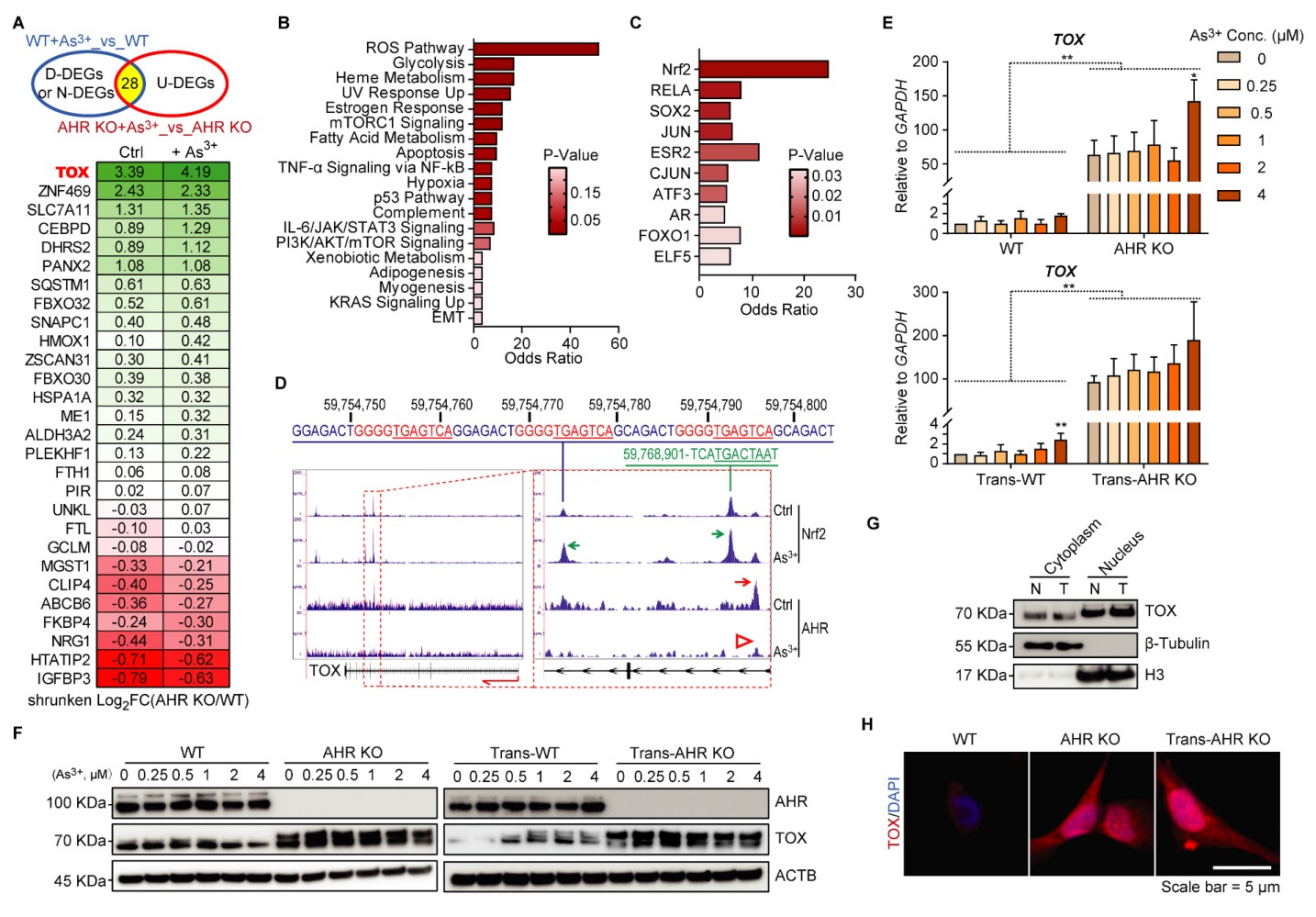
AHR represses TOX expression.** A**: Venn diagram illustrating 28 As³⁺-upregulated genes in AHR KO cells but not in WT cells, with their expression changes shown in the heatmap. D-DEGs: down-regulated differentially expressing genes; N-DEGs: No changes in expression; U-DEGs: upregulated differentially expressing genes. Data are presented as log2 fold changes between the indicated groups. **B**: GSEA of these 28 AHR-suppressed genes, highlighting significant impact on the ROS pathway in AHR KO cells after As³⁺ exposure. **C**: ChIP enrichment analysis (ChEA) of these genes, with Nrf2 identified as the most enriched transcription factor. **D**: ChIP-seq data showing that As³⁺ treatment enhances NRF2 binding to the TOX gene while reducing AHR binding. Two NRF2 binding peaks are located within intron 4 and intron 5 of the TOX gene (highlighted by red dashed boxes). A single AHR binding peak is positioned upstream of Nrf2 binding sites within intron 4. **E**: qPCR results of *TOX* expression in WT, AHR KO, Trans-WT, and Trans-AHR KO cells after 0-4 μM As³⁺ treatment for 6 h. Data are presented as mean ± SD, n = 5, **p < 0.01 vs. corresponding WT or Trans-WT. **F**: Immunoblots demonstrating the dose-dependent response of TOX levels in WT, AHR KO, Trans-WT and Trans-AHR KO cells following 0-4 μM As^3+^ treatment for 6 h. **G**: Cytoplasmic and nuclear fractionation showing moderate increase in TOX nuclear translocation in Trans-AHR KO cells (T) compared to AHR KO cells (N). **H**: Immunofluorescence of TOX in WT, AHR KO and Trans-AHR KO cells.

To uncover the initial molecular mechanisms underlying As³⁺-induced malignant transformation and the role of AHR, we performed RNA-seq on WT and AHR KO cells under basal and short-term (12 h) As³⁺ exposure. We focused on DEGs that were upregulated by As³⁺ in AHR KO cells but not in WT cells, identifying them as AHR-repressed genes to investigate AHR's regulatory role (**Figure 3A**). Several known Nrf2-target genes, such as ZNF469 32, SLC7A11 33, SQSTM1 34, and ALDH3A2 35 were significantly overexpressed in AHR KO cells compared to WT cells, suggesting an inhibitory effect of AHR on Nrf2 signaling. Additionally, HMOX1 36 and CEBPD 37, robust markers of Nrf2 activity, were upregulated by AHR knockout, with this effect becoming more pronounced upon As³⁺ treatment. These findings suggest that As³⁺ activates Nrf2 signaling, while AHR acts as a suppressor of Nrf2 activity. To further explore the functional consequences of AHR suppression, we conducted GSEA on these 28 AHR-repressed genes. The results revealed that the reactive oxygen species (ROS) pathway was significantly upregulated in AHR KO cells following As³⁺ exposure. Additionally, these genes were also enriched in cancer-related pathways, including glycolysis, hypoxia, adipogenesis, myogenesis, KRAS signaling, and EMT (**Figure 3B**). Interestingly, their regulation was not limited to Nrf2—previously identified in our study as an oncogenic driver in As³⁺-induced carcinogenesis 16—but also involved other stemness-related transcription factors, including SOX2, JUN, ATF3, and FOXO1 (**Figure 3C**). Of note, *TOX* was the only gene absent in WT cells but expressed exclusively in AHR KO cells, with further elevation following As³⁺ treatment (**Figure 3A**). Building on our previous findings that Nrf2 activation promotes As^3+^-induced malignancy in BEAS-2B cells, we further examined the antagonistic role of AHR on Nrf2 in this process. Integration of our ChIP-seq data for Nrf2 and AHR revealed binding peaks for both transcription factors within intron 4 of the TOX gene, rather than at the promoter, as typically expected. Further analysis identified a single AHR binding peak positioned upstream of the Nrf2 binding peaks. Notably, the Nrf2 binding peaks in intron 5 contain three conserved and identical Nrf2 binding elements (GGGGTGAGTCA) spanning a 60 bp region. Moreover, As³⁺ treatment enhanced Nrf2 binding to the TOX gene while reducing AHR binding (**Figure 3D**), suggesting that Nrf2 promotes, whereas AHR suppresses, *TOX* expression in As³⁺-induced carcinogenesis. This finding aligns with our previous observations 17. To further validate AHR's role in *TOX* regulation, qPCR analysis showed significantly elevated *TOX* mRNA levels in AHR KO cells compared to WT cells upon short-term (12 h) As³⁺ treatment, with a 50-fold increase in the TOX/GAPDH ratio (**Figure 3E**). A similar pattern was observed in the As^3+^-transformed WT (Trans-WT) and Trans-AHR KO cells (**Figure 3E**). Additionally, after treating WT, Trans-WT, AHR KO, and Trans-AHR KO cells with 0-4 μM As³⁺ for 6 h, TOX protein levels were significantly higher in AHR KO and Trans-AHR KO cells compared to their WT and Trans-WT counterparts, respectively (**Figure 3F**). Subcellular fractionation analysis revealed a moderate increase in TOX nuclear translocation in Trans-AHR KO cells compared to non-transformed AHR KO cells, indicating its activation and nuclear localization for functional execution (**Figure 3G**). These findings were further supported by immunofluorescence analysis of TOX, which demonstrated increased TOX abundance in nuclei of both AHR KO and Trans-AHR KO cells (**Figure 3H**). Notably, Trans-AHR KO cells exhibited more pronounced TOX nuclear translocation compared to AHR KO cells. Collectively, these results demonstrate that AHR functions as a repressive transcription factor, inhibiting TOX expression.

### Silencing *TOX* decreases As^3+^-induced malignant transformation potential in AHR KO cells

**Figure 4:**
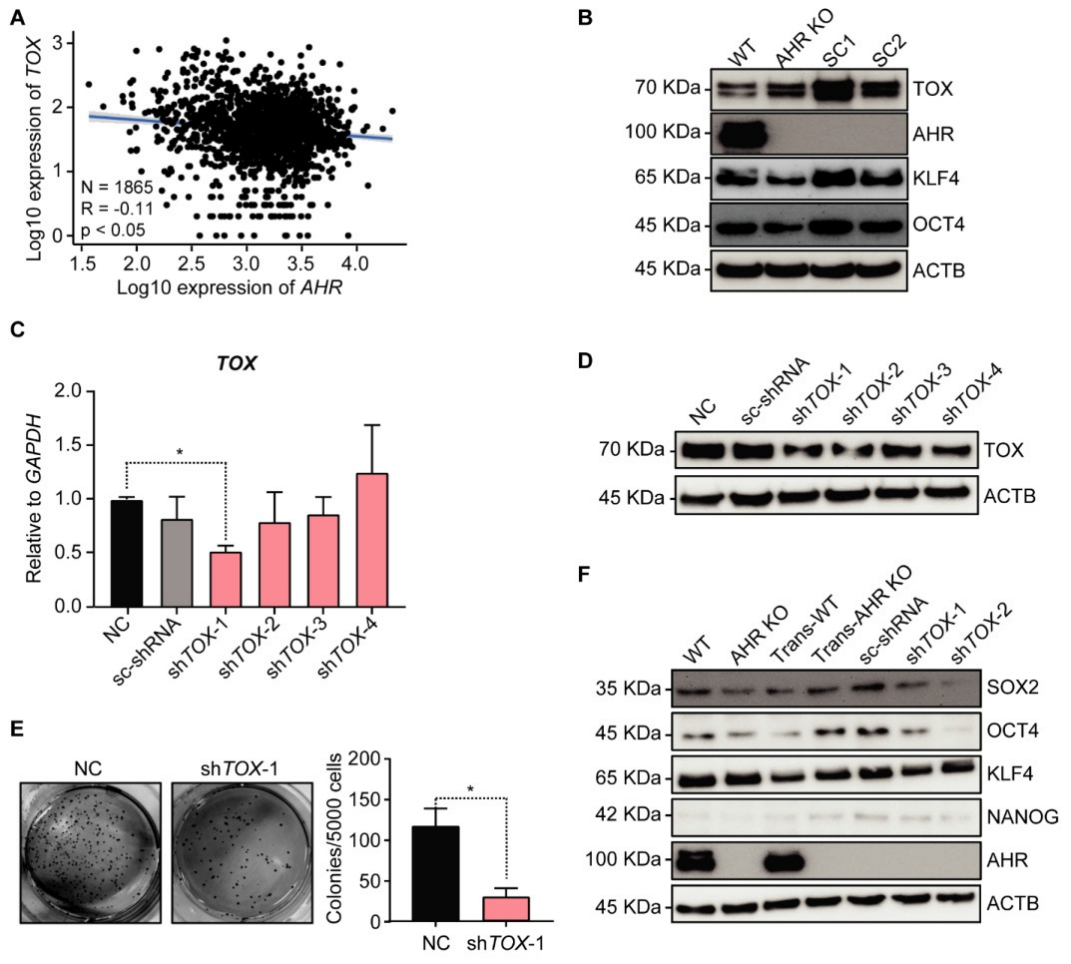
Silencing *TOX* reduces As^3+^-induced malignant transformation potential in AHR KO cells.** A**: Correlation between *AHR* and *TOX* expression using RNA-seq data from human lung cancer. **B**: Immunoblots of TOX, AHR, KLF4, OCT4, and ACTB in WT, AHR KO, and AHR KO-derived single clones (SC1 and SC2). **C**: qPCR results of *TOX* expression in Trans-AHR KO cells and Trans-AHR KO cells with *TOX* knockdown. Data are presented as mean ± SD, n = 5, *p < 0.05 vs. NC. NC: non-specific control. sc-shRNA: scramble shRNA. **D**: Immunoblots showing TOX expression in Trans-AHR KO cells and Trans-AHR KO cells with *TOX* knockdown. **E**: Representative images of whole-well soft agar colony formation in Trans-AHR KO cells and Trans-AHR KO cells with *TOX* knockdown, accompanied by colony count quantification. **F**: Immunoblots of SOX2, OCT4, KLF4, NANOG, AHR and ACTB in WT, AHR KO, Trans-WT, Trans-AHR KO and Trans-AHR KO cells with *TOX* knockdown.

To explore the role of TOX in carcinogenesis, we analyzed the correlation between AHR and TOX expression using RNA-seq data from human lung cancer, which revealed a negative correlation between AHR and TOX (**Figure 4A**). Given AHR's previously demonstrated tumor-suppressive role in lung cancer 17, TOX upregulation may contribute to As³⁺-induced malignancy. We then examined TOX protein levels in the transformed cells as represented by the individual clones from a soft agar assay using AHR KO cells, observing that TOX protein expression was significantly elevated in the clones compared to parental AHR KO cells (**Figure 4B**). Additionally, we observed notable upregulation of key stemness transcription factors, KLF4 and OCT4, essential for CSC formation, in the transformed subclones (SC1 & SC2). These findings suggest that TOX enhances anchorage-independent growth, a hallmark of oncogenic transformation. To test the hypothesis that TOX acts as a pro-oncogenic factor contributing to As^3+^-induced malignancy in AHR KO cells, we used lentiviral shRNAs to silence TOX in Trans-AHR KO cells. Knockdown efficiency was confirmed by qPCR and Western blotting, showing a substantial reduction in TOX at both mRNA and protein levels in the cells infected with shTOX-1 and shTOX-2 (**Figure 4C-D**). In a subsequent soft agar transformation assay, we found that silencing *TOX* significantly reduced colony formation in Trans-AHR KO cells (**Figure 4E**), underscoring TOX's essential role in promoting As³⁺-induced malignant transformation. Furthermore, *TOX* silencing led to a marked downregulation of SOX2 and OCT4 (**Figure 4F**). These results support our hypothesis that TOX functions as an oncogenic protein in As^3+^-induced carcinogenesis in lung bronchial epithelial cells.

### TOX acts as a pro-oncogenic factor in As^3+^-induced malignancy by interacting with stemness-related proteins

**Figure 5:**
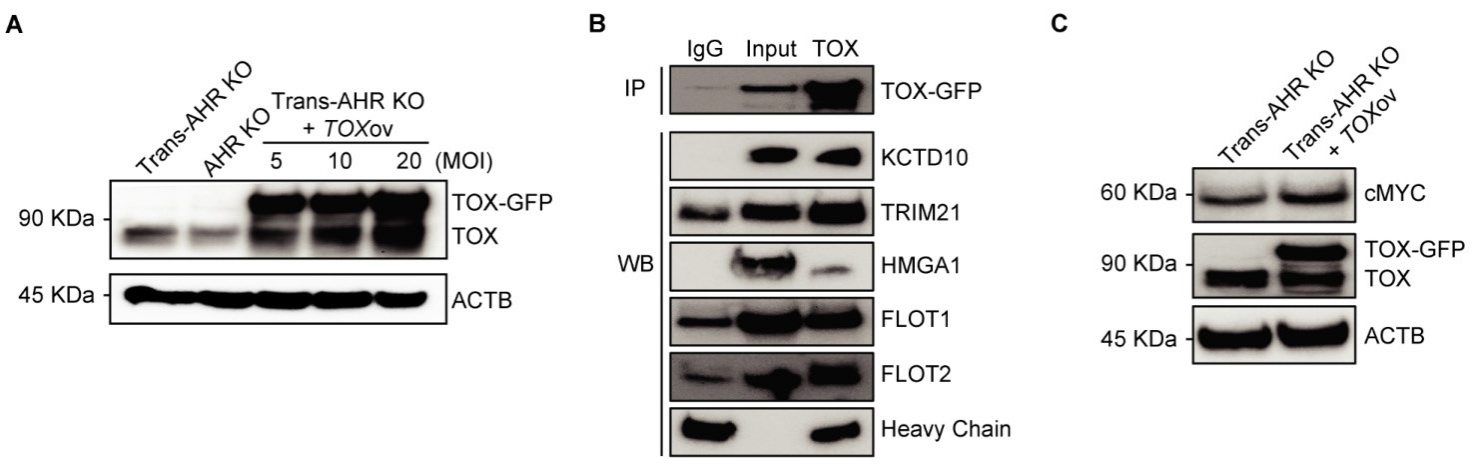
TOX enhances As^3+^-induced malignant transformation through interactions with stemness-related proteins. **A**: Immunoblots showing TOX expression in Trans-AHR KO, AHR KO, and AHR KO cells transfected with varying concentrations of GFP-tagged TOX lentivirus. **B**: Co-IP results showing strong binding between TOX and proteins such as KCTD10, TRIM21, HMGA1, FLOT1, and FLOT2. **C**: Immunoblots of cMYC, TOX-GFP, TOX and ACTB in Trans-AHR KO cells and Trans-AHR KO cells following *TOX* overexpression.

To elucidate the oncogenic role of TOX in As³⁺-induced malignancy, we overexpressed TOX conjugated with a GFP tag in transformed AHR KO cells using lentiviral vector, aiming to investigate whether the expression of exogenous TOX affects the expression of genes critical to carcinogenesis. As shown in **Figure 5A**, TOX protein levels were significantly increased following transfection, with TOX-GFP bands observed exclusively in TOX-overexpressing Trans-AHR KO cells. The band intensity correlated with the efficiency of overexpression. While the biological function of TOX protein in immune cells is well-documented, its role in non-immune cells remains to be understood. To gain insights into the functional activity of TOX, we performed preliminary proteomics analysis of TOX-GFP immunoprecipitates using LC-MS/MS technology. Due to detection limitations and the low specificity of available antibodies, considerable variations were observed in several attempts. However, proteins, such as KCTD10, TRIM21, HMGA1, FLOT1, and FLOT2, which interact with TOX, were frequently noted in these assays. To confirm these interactions, we conducted co-IP experiments and demonstrated binding between TOX and proteins like KCTD10, TRIM21, HMGA1, FLOT1, and FLOT2 (**Figure 5B**). Based on literature reviews, HMGA1, a chromatin-binding protein, has been linked to enhanced Wnt signaling in both intestinal stem cells and cancer stem cells 38, suggesting that TOX's oncogenic activity could involve chromatin interactions and stemness regulation. TOX's interaction with FLOT proteins also hints at a role in vesicular trafficking and membrane translocation processes 39. Further Western blot analysis demonstrated increased c-MYC levels following TOX overexpression in Trans-AHR KO cells (**Figure 5C**). Taken together, these findings indicate that elevated TOX expression promotes CSC formation during As³⁺-induced carcinogenesis by interacting with proteins that support stemness and oncogenic pathways.

### Regulation of TOX stability via KCTD10/TRIM21 complex-mediated ubiquitin-proteasome pathway

**Figure 6:**
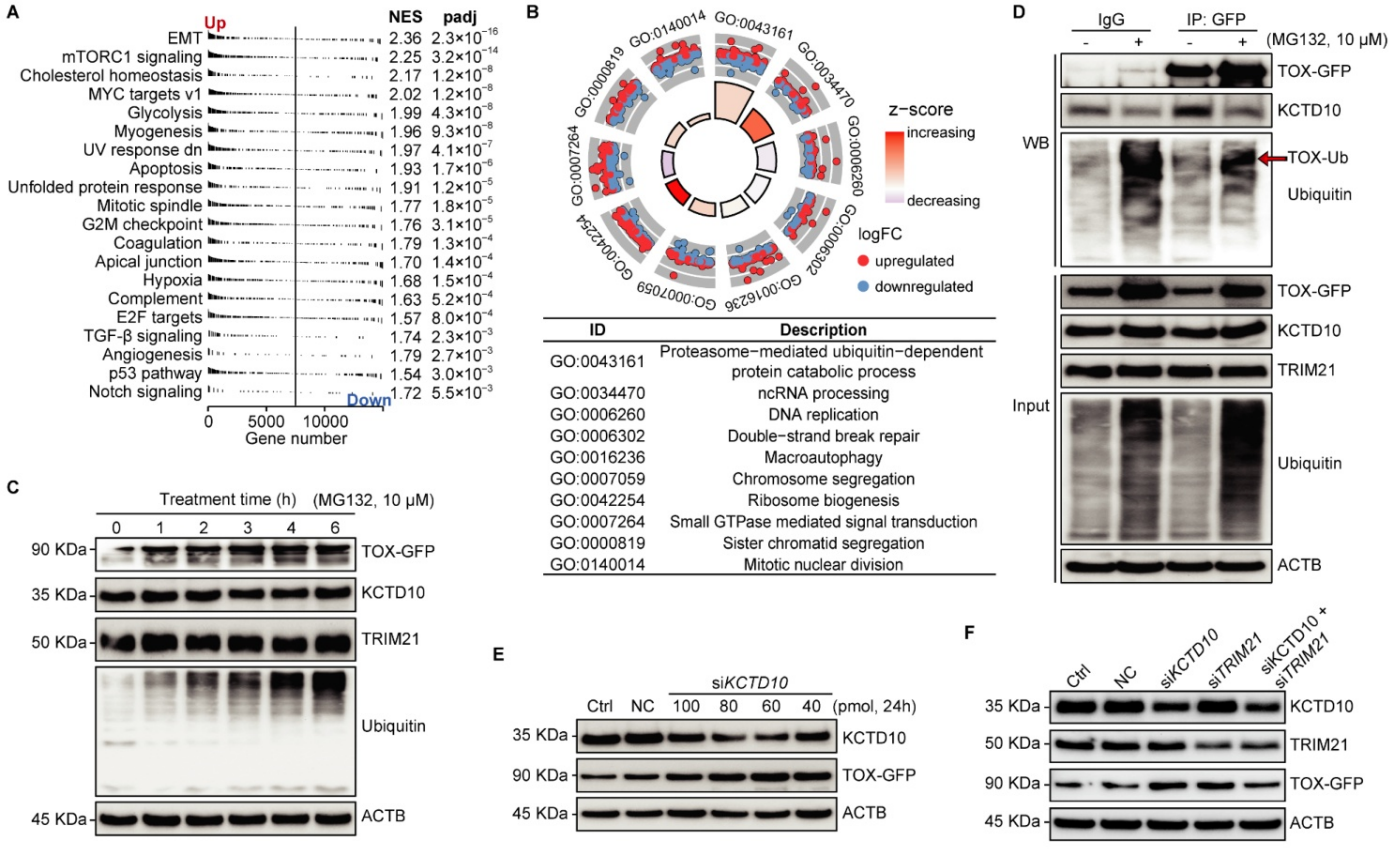
KCTD10/TRIM21 complexes regulate TOX stability via the ubiquitin-proteasome pathway. **A**: GSEA from the Hallmark collection showing the Top 20 pathways affected by *TOX* overexpression in Trans-AHR KO cells (NES: normalized enrichment score). **B**: Biological process analysis highlighting the top 10 biological processes enriched by genes upregulated in Trans-AHR KO cells with *TOX* overexpression. **C**: Time-course analysis of MG-132 treatment on TOX-GFP, KCTD10, TRIM21, Ubiquitin, and ACTB abundance in Trans-AHR KO cells with *TOX* overexpression. **D:** Western blot analysis of immunoprecipitated samples showing the impact of MG-132 on protein binding between KCTD10, TRIM21 and TOX. **E**: Immunoblots showing the effect of *KCTD10* silencing on TOX-GFP levels in Trans-AHR KO cells with *TOX* overexpression. **F**: Immunoblots showing the impact of silencing *KCTD10*, *TRIM21* or both on TOX-GFP levels in Trans-AHR KO cells with *TOX* overexpression. NC: non-specific control.

To additionally reveal the role of TOX in the tested cells, we conducted RNA-seq to uncover molecular events affected by *TOX* overexpression in Trans-AHR KO cells. GSEA indicated that *TOX* overexpression significantly activates cancer-related pathways, including EMT, glycolysis, myogenesis, and hypoxia (**Figure 6A**). GO analysis of TOX-upregulated genes showed involvement in proteasome-mediated, ubiquitin-dependent protein catabolism (**Figure 6B**). As illustrated in **Figure 5B**, KCTD10 acts as an adaptor protein for E3 ubiquitin ligases 40, while TRIM21 is a RING finger E3 ubiquitin ligase within the SKP1-CUL1-F-box (SCF) complex 41. TOX binds with KCTD10 and TRIM21, suggest that TOX may be involved in the ubiquitination and proteasomal degradation of other proteins, potentially including regulation of its own stability. To test this, we treated TOX-GFP cells with the proteasome inhibitor MG132 over a time course and observed a time-dependent accumulation of TOX following MG132 treatment compared to untreated cells (**Figure 6C**). MG132 did not affect KCTD10 or TRIM21 expression levels, which we confirmed via Western blotting with an anti-ubiquitin antibody, demonstrating specificity in proteasome inhibition. To assess whether KCTD10 functions as an adaptor influencing TOX stability, we performed a pull-down of TOX-GFP in cells treated with or without MG132. We found that MG132 treatment significantly reduced KCTD10 binding to TOX, indicating that KCTD10 actively contributes to TOX degradation (**Figure 6D**). Furthermore, varying concentrations of siRNA-mediated *KCTD10* knockdown showed that TOX stability is indeed dependent on KCTD10 levels (**Figure 6E**). Notably, knockdown efficiency was higher at 60 and 80 pmol siRNAs compared to 100 pmol. Pearson's correlation analysis further confirmed a strong negative correlation between KCTD10 and TOX-GFP levels (correlation coefficient: -0.813, *p* < 0.05), reinforcing the role of KCTD10 in regulating TOX stability (**Figure 6E**). To determine whether KCTD10 and TRIM21 function as a complex, we knocked down KCTD10, TRIM21, or both using siRNAs and examined their effects on TOX stability. Individual knockdown of either *KCTD10* or *TRIM21* significantly increased TOX levels (**Figure 6F**), indicating that both proteins contribute to TOX degradation. Interestingly, simultaneous knockdown of both *KCTD10* and *TRIM21* did not further elevate TOX expression (**Figure 6F**), suggesting the involvement of additional factors in TOX regulation. Collectively, these findings highlight the role of the KCTD10/TRIM21 complex in controlling TOX stability via the ubiquitin-proteasome system.

## Discussion

Environmental As³⁺ exposure remains a major public health concern in the U.S. due to its widespread prevalence and potent carcinogenic effects in humans 42. AHR, a critical mediator in the early response to chemical exposures, plays a complex role in the carcinogenesis of various agents 43. Previous studies suggest that AHR exhibits tumor-suppressive effects in As³⁺-induced carcinogenesis 17. Our preliminary data reveal that As³⁺ treatment reduces AHR binding to genes involved in key oncogenic signaling pathways, correlating with increased expression of these AHR-target genes in the human bronchial epithelial cell line BEAS-2B 15. These results support AHR's protective role against As³⁺-induced transformation. Nevertheless, the precise role of AHR in As³⁺-driven carcinogenesis remains uncertain, underscoring the need to elucidate the molecular mechanisms through which AHR modulates cancer initiation and progression.

AHR was initially identified as a transcription factor mediating responses to polycyclic aromatic hydrocarbons, essential for regulating xenobiotic-metabolizing enzymes, especially cytochrome P450 enzymes 44. In its inactive state, AHR is sequestered in the cytoplasm. Upon ligand binding, AHR translocates to the nucleus, where it heterodimerizes with the aryl hydrocarbon receptor nuclear translocator (ARNT) 45. This AHR-ARNT complex then binds to xenobiotic response elements (XRE) within gene promoter or other regions, regulating a wide range of target genes involved in angiogenesis, hematopoiesis, metabolism, cell motility, and immune modulation 46. Recent research has highlighted AHR's complex dual role in carcinogenesis, where it can act as either a tumor promoter or suppressor depending on cellular context. For instance, a study by Ouyang et al. demonstrated that AHR acts as an upstream transcription factor, with UCHL3 functioning as a deubiquitinating enzyme that stabilizes AHR 18. This stabilization was shown to increase the binding of AHR to the promoter regions of the stemness genes such as *ABCG2*, *KLF4*, and *cMYC*, promoting tumor growth and enhancing lung cancer stem-like properties. In contrast, findings from Cheng et al. indicated a strong negative correlation between the AHR and OCT4 expression in embryonic stem cell H1 and embryonal carcinoma cell NCCIT undergoing retinoic acid (RA)-induced differentiation 21. This suggests that AHR may exert opposing effects in different cellular environments, further complicating its role in cancer biology. Our previous study also showed that AHR is typically enriched on various oncogenic genes, in addition to the well-established phase I/II enzymes, including genes involved in TGFβ and Nrf2 signaling pathways, as well as several known oncogenes 15. However, As³⁺ exposure significantly reduced AHR binding to these genes, resulting in increased expression of these target genes. These findings highlight the intricate and context-dependent nature of AHR's involvement in cancer, underscoring the need for further investigation into its dual role and potential as a therapeutic target.

TOX has been identified as a critical regulator in both immune cell differentiation and neural stem cell commitment, playing a pivotal role in corticogenesis 47. Studies indicate that TOX maintains the neural stem cell pool by inhibiting the transition from proliferative progenitors to differentiating ones during cortical development 48. In addition to its function in progenitor cells, TOX promotes neurite outgrowth in newly formed neurons migrating toward the cortical plate, likely by regulating critical genes involved in neural stem cell fate, such as *SOX2*, *EOMES*, and *ROBO2* 48. In the immune system, TOX functions as a developmental checkpoint and regulates thymocyte positive selection during T cell lineage commitment. It is essential for the maturation of various T cell subsets, including CD4^+^ helper T cells, CD8^+^ cytotoxic T cells, regulatory T cells, and CD1D-dependent natural killer T (NKT) cells 49, 50. Under chronic antigen stimulation, TOX modifies T cell development by promoting the generation of exhausted T cells while inhibiting effector and memory T cell programming 51. It may regulate genes encoding inhibitory receptors like *PDCD1*, inducing an exhaustion program that prevents overstimulation and activation-induced cell death in T cells. While the cancer-related roles of TOX are still not fully understood, recent studies have shown that TOX expression or mutations are deregulated in several diseases, particularly in malignancies like lung, breast, and gastric cancers, as well as lymphomas and leukemia. A study by Lobbardi et al. identified TOX as a collaborating oncogenic driver in T-cell Acute Lymphoblastic Leukemia (T-ALL) 52. This research revealed that TOX accelerates the onset of T-ALL by promoting aberrant DNA repair, leading to increased genomic instability and expansion of the transformed clone pool, emphasizing its role in the progression of this malignancy.

Emerging evidence indicates that High Mobility Group A1 (HMGA1) portends poor clinical outcomes 38, 53. By competing with histone H1 for DNA binding, HMGA1 modifies DNA conformation, widens the minor groove, and facilitates the recruitment of transcription factor complexes and chromatin modifiers, thereby influencing gene expression and driving oncogenic transcriptional networks in cancer and stem cells 54. In this study, we identified a direct interaction between TOX and HMGA1 in As³⁺-transformed AHR KO cells, suggesting that TOX may stabilize HMGA1 and enhance its oncogenic activity. This finding further supports TOX's role in regulating stemness-related gene transcription and underscores its broader implications in cancer progression.

FLOT1 is a scaffold protein of lipid rafts and has been found to be upregulated in various cancers, making it a potential target for cancer therapy 55. Previous studies have shown that FLOT1 promotes gastric cancer progression and metastasis by interacting with BCAR1, specifically regulating its phosphorylation and translocation 56. Additionally, the sumoylation of FLOT1 enhances EMT and cancer metastasis by inhibiting the degradation of Snail, a key transcription factor that regulates EMT-related gene expression 57. One of the most critical mechanisms by which FLOT1 facilitates EMT is through the formation of lipid rafts, which play a pivotal role in this process. During EMT, epithelial markers such as E-cadherin are downregulated, whereas mesenchymal markers such as N-cadherin and integrins are upregulated. Lipid rafts mediate the internalization and degradation of E-cadherin, thereby reducing cell adhesion and promoting EMT 58. In our study, the TOX interactome results revealed an interaction between TOX and FLOT1/FLOT2, further supporting the oncogenic role of TOX in EMT regulation.

Protein homeostasis is essential for various cellular functions, with the ubiquitin-proteasome system and lysosomal proteolysis serving as the two primary pathways for protein degradation. In this study, we identified KCTD10 as a substrate recognition receptor that regulates TOX stability through the ubiquitin-proteasome system. KCTD10 has been reported to target multiple substrates, including KCTD13, RhoB, and Notch1 59-61. Our findings demonstrate that TOX degradation occurs in a KCTD10-dependent manner, likely through the formation of a KCTD10-Cullin-3 E3 ubiquitin ligase complex. Additionally, our data suggests that KCTD10 interacts with TRIM21, another E3 ubiquitin ligase, to facilitate TOX degradation. However, the simultaneous knockdown of both *KCTD10* and *TRIM21* did not further increase TOX expression, indicating the involvement of additional proteins in this regulatory complex—potentially proteins that interact weakly or indirectly with TOX. Further investigation is required to elucidate the composition of this complex and its role in As³⁺-induced carcinogenesis.

**Figure 7:**
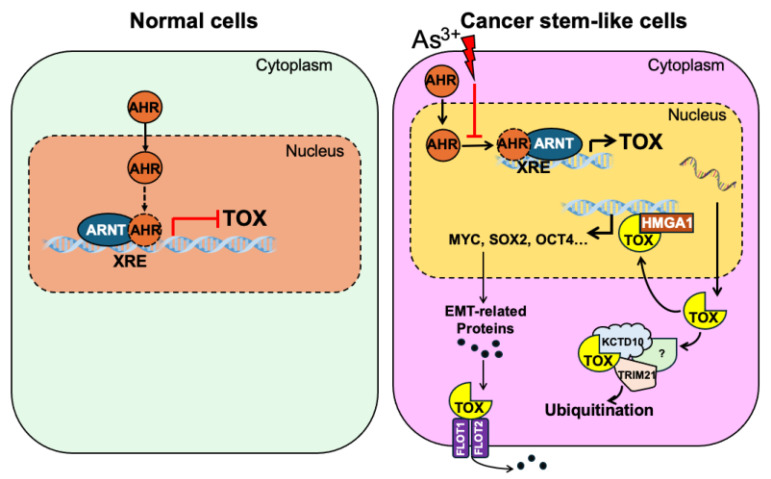
As^3+^ disrupts AHR's repressive effect on TOX gene transcription, promoting malignant transformation and the potential emergence of cancer stem-like cells (CSCs). This disruption leads to elevated TOX expression, which directly interacts with HMGA1, potentially amplifying its oncogenic effects. Additionally, the TOX protein may associate with KCTD10 and TRIM21—two proteins involved in ubiquitination and proteasomal degradation—either regulating its own stability or influencing broader protein turnover. Furthermore, TOX's interactions with FLOT1 and FLOT2 may enhance their roles in caveolae or caveolae-like vesicle formation, facilitating cross-membrane transport—an essential process in cancer cell metastasis.

In this study, we provide compelling evidence that AHR suppresses transformation as well as the possible formation of cancer stem-like cells (CSCs) induced by low-dose As³⁺ exposure through the transcriptional repression of *TOX* **(Fig. 7)**. Our results demonstrate that *TOX* knockdown inhibits As³⁺-induced transformation, while *TOX* overexpression upregulates the CSC-associated transcription factor MYC. Further analysis indicated direct interaction of TOX with proteins such as KCTD10, TRIM21, HMGA1, FLOT1, and FLOT2, which may influence TOX's stability and activity **(Fig. 7)**. Together, these findings suggest that TOX functions as an oncogenic factor and that AHR's tumor-suppressive role is, at least in part, mediated by repressing TOX expression. Notably, this study is the first to highlight TOX's oncogenic role outside of immunological contexts, providing new insights into the molecular mechanisms underlying As³⁺-induced carcinogenesis.

## Conclusion

This study offers novel insights into the complex regulation of TOX by AHR, highlighting that AHR functions as a tumor suppressor by repressing TOX transcription. However, there are several limitations to consider. Firstly, the TOX interactome was established using pull-down of overexpressed TOX-GFP, a non-native protein, which may result in the artificial binding of other proteins. Secondly, while co-immunoprecipitation revealed interactions of TOX with KCTD10, TRIM21, HMGA1, FLOT1, and FLOT2, the precise contribution of these interactions to As³⁺-induced malignant transformation remains unclear. Nevertheless, our findings indicate that TOX acts as an oncogenic factor in As³⁺-induced malignant transformation and CSC generation. Further research is needed to fully elucidate the molecular mechanisms underlying TOX's role in cancer, its interactions with AHR, and its potential as a therapeutic target in As³⁺-related carcinogenesis and other malignancies. These findings pave the way for further exploration of the AHR-TOX axis and its therapeutic potential in cancer treatment.
